# Lattice–Gas–Automaton Modeling of Income Distribution

**DOI:** 10.3390/e22070778

**Published:** 2020-07-17

**Authors:** Lijie Cui, Chuandong Lin

**Affiliations:** 1 School of Labor Economics, Capital University of Economics and Business, Beijing 100070, China; cuilijie@cueb.edu.cn; 2 Sino-French Institute of Nuclear Engineering and Technology, Sun Yat-Sen University, Zhuhai 519082, China; 3 Key Laboratory for Thermal Science and Power Engineering of Ministry of Education, Department of Energy and Power Engineering, Tsinghua University, Beijing 100084, China

**Keywords:** lattice gas automaton, income distribution, Matthew effect, income tax, charity

## Abstract

A simple and effective lattice–gas–automaton (LGA) economic model is proposed for the income distribution. It consists of four stages: random propagation, economic transaction, income tax, and charity. Two types of discrete models are introduced: two-dimensional four-neighbor model (D2N4) and D2N8. For the former, an agent either remains motionless or travels to one of its four neighboring empty sites randomly. For the latter, the agent may travel to one of its nearest four sites or the four diagonal sites. Afterwards, an economic transaction takes place randomly when two agents are located in the nearest (plus the diagonal) neighboring sites for the D2N4 (D2N8). During the exchange, the Matthew effect could be taken into account in the way that the rich own a higher probability of earning money than the poor. Moreover, two kinds of income tax models are incorporated. One is the detailed taxable income brackets and rates, and the other is a simplified tax model based on a fitting power function. Meanwhile, charity is considered with the assumption that a richer agent donates a part of his income to charity with a certain probability. Finally, the LGA economic model is validated by using two kinds of benchmarks. One is the income distributions of individual agents and two-earner families in a free market. The other is the shares of total income in the USA and UK, respectively. Besides, impacts of the Matthew effect, income tax and charity upon the redistribution of income are investigated. It is confirmed that the model has the potential to offer valuable references for formulating financial laws and regulations.

## 1. Introduction

Econophysics is a heterodox interdiscipline where economics and finance problems are solved with statistical physics methods, usually including uncertainty or stochastic processes and nonlinear dynamics [1,2,3,4]. As a central concern of economic theory and economic policy, distributions of money, wealth, and income have been widely studied due to their practical importance in human society [5,6,7,8,9,10,11,12]. One of the main assumptions in econophysics is that an economic system reaches its equilibrium state after a period of relaxation process. For an equilibrium economic system, money could be treated as a conserved quantity during a certain period of time, because the government should maintain money constant to avoid inflation or deflation phenomenon in the realistic society [13]. Analogous to statistical physics, the total money and average money of individual agents in an economic market are equivalent to the energy and temperature in a physical system. Actually, the income distribution of agents in a closed system could be expressed by an exponential Boltzmann–Gibbs function, which is in analogy with the energy distribution in statistical physics [3,13,14]. Using tax and census data in the USA, Dr$\overset{˘}{a}$gulescu and Yakovenko demonstrated that the individual income distribution is exponential [14]. Later, Newby et al. revealed that the income distribution in the US is characterized by the two groups corresponding to the Boltzmann–Gibbs and Pareto functions [15]. In statistical physics, the Boltzmann–Gibbs distribution is associated with the maximum entropy, and is a sufficient and necessary condition for the equivalence between the Gibbs–Shannon entropy and the thermodynamic entropy [16,17]. In economics, the Boltzmann–Gibbs distribution could also be used to allocate permits in emissions trading and describe the most probable, natural, and unbiased distribution of emissions permits among multiple countries [18].

In recent years, with the rapidly improving computational facilities and algorithms, numerical simulation has achieved great success in econophysics, and the income distribution has been mimicked and studied with a series of effective methods [19,20,21,22,23,24,25]. In 2000, a statistical economic model was considered for a market where the total money of a fixed number of agents is constant, and the impact of the saving propensity of the agents upon the equilibrium probability distribution of money was studied [19]. In 2008, Bargain and Callan proposed a methodology based on counterfactual tax-benefit simulations to evaluate the influence of tax-benefit policy changes upon income distribution over time [20]. In 2013, Cerd$\overset{´}{a}$ et al. proposed an econophysics model based on a lattice–gas–automaton (LGA) for income distribution under charity strategies (i.e., a richer agent voluntarily gives money to the one without money) [21]. In 2015, Dafermos and Papatheodorou developed a benchmark stock-flow consistent model linking functional with personal income distributions [26]. In 2016, Rey et al. performed Monte Carlo simulations and examined the properties of tests for spatial effects in Discrete Markov chain models of regional distribution dynamics [22,23]. In 2019, Alves and Monteiro developed a spatial evolutionary version of the ultimatum game as a toy model of income distribution, where the money does not disappear when an offer is not taken [24]. In 2020, Tian and Liu proposed an inhomogeneous agent-based model to study the emergence of income inequality, where individuals with varied qualities work, consume and invest [25].

As a type of cellular automata, the LGA is almost the simplest among the aforementioned effective models [27,28,29]. The LGA method was pioneered by the Hardy–Pomeau–de Pazzis model in 1973 [28]. This model aimed to simulate the Navier–Stokes (NS) equations but the lack of rotational invariance made it highly anisotropic [28]. In 1986, the Frisch–Hasslacher–Pomeau model was proposed as a successful LGA where the tensor of rank four formed from products of the lattice vectors is isotropic [30]. The finding of the lattice symmetry condition started an avalanche of LGA models [31]. At present, the LGA has been developed to simulate some phenomena in hydrodynamics [30], chemistry [32,33], electromagnetics [34], thermoacoustics [35], economics [21], etc. In fact, the basic idea of LGA is that different microscopic behaviors can lead to similar macroscopic performance and artificial particles move on lattices with interactions which conserve physical quantities such as mass and momentum [31]. The microdynamics of such an artificial world should be as simple as possible for the sake of high computational efficiency [31,36]. Furthermore, the LGA was the precursor to the lattice Boltzmann method (LBM) [36,37,38,39,40,41] and discrete Boltzmann method (DBM) [42,43,44,45,46,47,48]. In a broad sense, the LGA and its offspring (LBM/DBM) are regarded as mesoscopic kinetic models with the following similar virtues. (i) It is easy to code and implement the program due to their simple schemes. (ii) Because of the local character of the update rules, they are well suited for massively parallel computers. (iii) These models are capable of simulating systems with complex geometry by employing appropriate boundary conditions.

In this work, the LGA is further developed for simulations of income distribution in an economic society. The rest of the paper is organized as follows. In Section 2, we introduce the details of the LGA economic model. The model is then validated in Section 3. Finally, conclusions and discussions are given in Section 4.

## 2. Lattice–Gas–Automaton Economic Model

The current LGA economic model describes an artificial society where agents perform monetary transactions when they encounter each other after random movements. Individuals are regarded as ideal gas molecules, and the internal energy of particles denotes the money of agents. The particle system is a coarse-graining model of the human society, and it evolves in a two-dimensional computational domain, which seems to represent a human territory in a natural way.

### 2.1. Computational Domain

**Figure 1:**
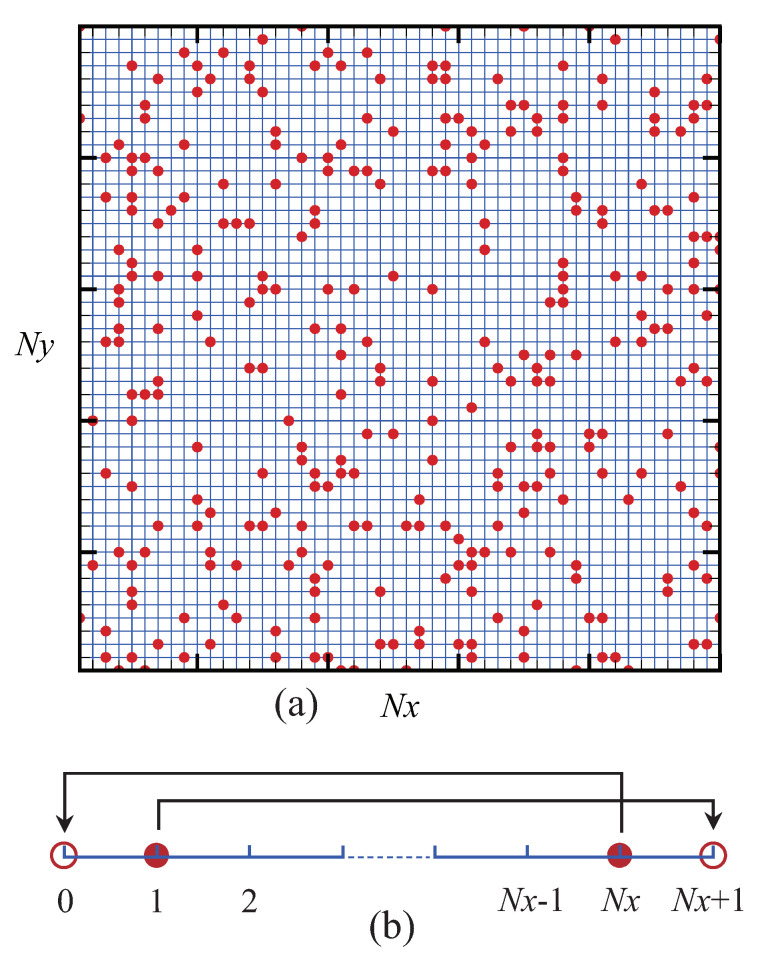
Sketch of: the initial configuration (**a**); and the boundary conditions (**b**).

Figure 1 illustrates the sketch of an initial configuration (Figure 1a) and boundary conditions (Figure 1b). Agents (particles) are randomly scattered over a rectangular lattice with sites $N_{x}\times N_{y}$. Let $A_{i}$ indicate the agent possessing money $m_{i}$, with the subscript i=1, 2, ⋯, $N_{a}$, where $N_{a}$ denotes the total number of agents. The initial amount of money for each agent is given as $m_{0}$. In addition, the periodic boundary conditions are imposed on the computational domain. To be specific, in the horizontal direction, the agent on the left ghost site $A_{0}$ is the same as $A_{N_{x}}$, while the individual on the right ghost site $A_{N_{x}+1}$ is identical to $A_{1}$. The vertical boundaries are treated in a similar way.

### 2.2. Random Propagation

**Figure 2:**
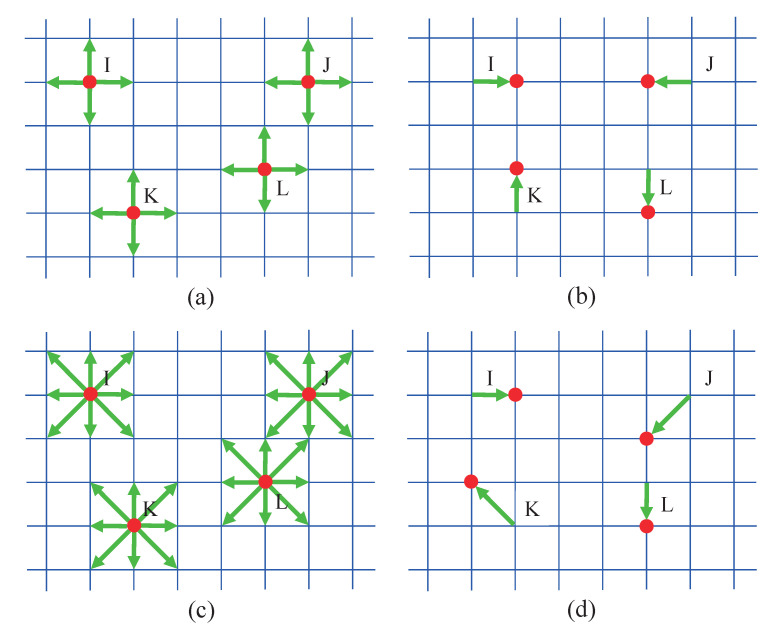
Agents’ random-walk process: (**a**) before propagation in D2N4; (**b**) after propagation in D2N4; (**c**) before propagation in D2N8; and (**d**) after propagation in D2N8.

Figure 2 displays the random-walk of agents on a rectangular lattice. It shows a simple dynamical movement: Time advances in discrete temporal steps Δt=1, and space is divided by spatial steps Δx=1 and Δy=1 in the horizontal and vertical directions, respectively. For each step, an agent either remains motionless (with probability $1-P_{m}$) or travels to its neighboring empty sites (with probability $P_{m}$) randomly. In the program, a random function is used to generate a random number *R* within the range from 0 to 1. If $R\leq P_{m}$, then the agent may move; otherwise, it does not move. Meanwhile, the model obeys the exclusion principle that prevents multiple particles from reaching the same site. Two types of velocity models are employed: two-dimensional four-neighbor model (D2N4) [21] and D2N8. For the former, an agent can only move to one of its four neighboring sites, which has not been occupied by other individuals (see Figure 2a,b), while, for the latter, the agent may travel to not only its nearest sites, but also the second nearest sites diagonally (see Figure 2c,d).

### 2.3. Economic Transaction

**Figure 3:**
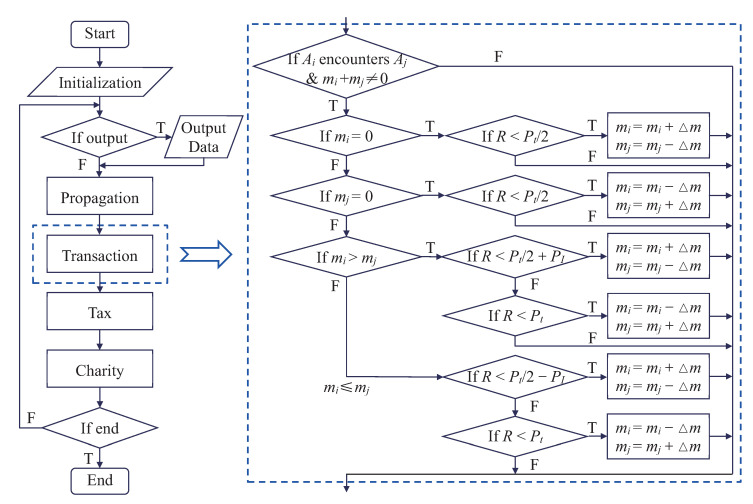
Program flow chart.

After random-propagation, the next phase is the economic transaction, which is the core module of the LGA economic model (see Figure 3). For the sake of simplification, a proper model is proposed to simplify the dynamics of the economic transaction. Specifically, an economic transaction takes place (with probability $P_{t}$) when two agents are located in neighboring sites. There are four and eight neighboring sites for D2N4 and D2N8, respectively. Here, the possibility of debt is not taken into account, i.e., $m_{i}\geq 0$. Consequently, there are four cases as follows.

Case I: $m_{i}=m_{j}=0$. Two agents without money cannot have an economic transaction.

Case II: $m_{i}=0$ and $m_{j}\neq 0$. An agent without money can only win or stay unchanged during an economic transaction.

Case III: $m_{i}\geq m_{j}>0$. There is a probability $P_{t}/2+P_{I}$ that the agent $A_{i}$ wins money, and a probability $P_{t}/2-P_{I}$ that the agent $A_{i}$ loses money.

Case IV: $m_{j}>m_{i}>0$. The probability is $P_{t}/2-P_{I}$ for $A_{i}$ earning money, and $P_{t}/2+P_{I}$ for $A_{i}$ losing money.

The amount of money transferred from one agent to the other is set as a fixed value, Δm. It should be noted that, with increasing Δm, the computing time decreases but the numerical results become less accurate. In this work, with the consideration of both computational efficiency and numerical accuracy, the money interchange is chosen as $\Delta m=m_{0}/100$ in each transaction process.

In addition, the parameter $P_{I}$ in the last two cases is utilized to control the inequality between the richer and poorer agents due to the Matthew effect [49,50]. In practice, the rich usually have a higher capability (more opportunities) to earn money than the poor. Namely, the rich become richer and the poor become poorer [50]. The range of the inequality parameter is $0\leq P_{I}\leq P_{t}/2$, and the current LGA reduces to a previous model [21] in the case $P_{I}=0$.

It is worth emphasizing that, at this point, the LGA economic model could recover the main features of the income distribution in a completely free market with conservative money. In practice, economic factors could be added to the model to increase its usefulness, such as the tax strategy, charity regulation, and other financial policies. The modules of income tax and charity are introduced in the next two subsections.

### 2.4. Income Tax

In fact, to reduce income inequality and improve economic system, one of the most effective measures is to formulate and implement reasonable tax regulations and policies. In this phase, the income tax is taken into consideration with two methods. One is to incorporate the detailed taxable income brackets and rates directly and the other is to employ a simplified tax model based on a fitting power function (see Appendix B). The former is straightforward, and the latter is introduced as below.

An agent $A_{i}$ should pay a tax if he earns money and his money $m_{i}$ is greater than a critical point $m_{tax}$, and the tax liability $\Psi_{i}$ takes the form

(1) $$\Psi_{i}=\psi_{i}\Delta m,$$

(2) $$\psi_{i}={\left(\frac{m_{i}}{m_{max}}\right)}^{\omega }\psi_{max},$$

where $\psi_{i}$ denotes the average tax rate, $m_{max}$ represents the maximum individual income, $\psi_{max}$ corresponds to the maximum tax rate in the case $m_{i}=m_{max}$, and the superscript ω is an empirical parameter which can be obtained by the fitting function of a realistic tax regulation. Otherwise, the agent is not taxed, i.e, $\Psi_{i}=0$.

Clearly, the post-tax individual income becomes

(3) $$m_{i}\leftarrow m_{i}-\psi_{i},$$

where “A←B” means “the variable *A* is updated with the value of *B*” in the program. The total amount of tax revenue is $\Phi_{t}=\sum_{i}\Psi_{i}$, which will be shared by all individuals equally. In other words, the money of each agent is then updated as

(4) $$m_{i}\leftarrow m_{i}+\frac{\Phi_{t}}{N_{a}}.$$

It can be found from Equations (3) and (4) that the total money is conserved and the income of the poorer (richer) agent is increased (decreased) by the tax policy.

### 2.5. Charity

In the stage of charity, money is redistributed from richer agents to poorer ones [51,52]. In this part, a simple charitable process is considered as follows. A richer agent whose money is greater than the threshold value $m_{r}$ donates a part of his income $m_{c}$ to charity (with probability $P_{c}$) when his money increases during an economic transaction. All charitable donations are distributed to the poorer agents whose income is below the poverty line $m_{p}$. The recipient is poorer than the donor, hence $m_{r}>m_{p}$.

In particular, a rich agent’s money changes as

(5) $$m_{i}\leftarrow m_{i}-m_{c},$$

if he makes a contribution to charity. Given the sum of contributions $\Phi_{c}$, a poor agent’s income is updated as

(6) $$m_{i}\leftarrow m_{i}+\frac{\Phi_{c}}{N_{p}},$$

if he receives donations. Here, $N_{p}$ represents the number of poor agents in need.

In sum, there are four key stages, namely, random propagation, economic transaction (with the Matthew effect), income tax, and charity, in the main loop of the program. The last three phases make competitive impacts on the distribution of individual income. Specifically, the economic transaction leads to income differences, and the Matthew effect exacerbates the disparity of wealth between the rich and poor, while the income tax and charity narrow the income gap. Furthermore, the LGA economic model is very simple, and it could be coded easily (see Figure 3). The program is written in Fortran 90 in this work.

## 3. Verification and Validation

In this section, two types of markets are considered as benchmarks to verify and validate the current LGA economic model. The first class is an ideal free market without any influence of human factors. Both D2N4 and D2N8 are employed, and all simulations give satisfactory results of the income distribution. The second one is a realistic social market under conditions of the Matthew effect, tax regulation, and charity. The shares of total income in the United States of America (USA) and United Kingdom (UK) are successfully mimicked. Besides, we investigate the individual impacts of the Matthew effect, income tax, and charity upon the redistribution of income in another subsection.

### 3.1. Income Distribution in the Free Market

The LGA economic model is used to mimic a particle system that represents an economic society with money conservation. First, let us consider a completely free market without the Matthew effect, income tax, or charity, i.e., $P_{I}=0$, $\psi_{c}=0$, and $m_{c}=0$. There are $N_{a}=600$ agents randomly scattered on a $N_{x}\times N_{y}=50\times 50$ square lattices. Each agent has been initialized to own money $m_{0}=4$. The probabilities of a random propagation and an economic transaction are $P_{m}=0.8$ and $P_{t}=0.7$, respectively.

**Figure 4:**
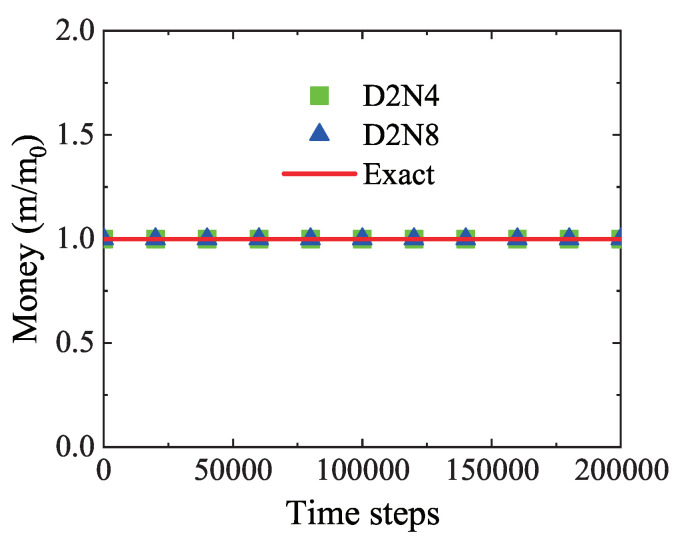
Money conservation in the evolution of an economic system.

To validate that the principle of money conservation is obeyed, Figure 4 exhibits the average value of money versus time steps. The squares stand for D2N4 results, the triangles for D2N8 results, and the solid line for the exact value $m_{0}=4$. Obviously, both D2N4 and D2N8 give simulation results which coincide with the exact one during the whole life-cycle of the economic system. That is to say, the money is conserved in the current model.

It is worth mentioning that the model has a high calculation efficiency. To conduct the simulation with $2\times 10^{5}$ time steps in Figure 4, it only takes 138 s for D2N4 and 312 s for D2N8. Here, the computational facility is a personal computer with Intel(R) Core(TM) i7-8750H CPU @ 2.20 GHz and RAM 16.0 GB. Although it takes longer computing time to run one time step by using D2N8 than D2N4, it requires fewer time steps to obtain an equilibrium state by using D2N8 than D2N4. In the above simulations, it takes about 46,000 time steps for the economic system to start to reach the equilibrium state by using D2N4, and needs about 20,000 time steps by using D2N8. In other words, it takes almost the same computation time to obtain an equilibrium state by using either of them. It is reasonable, because there are more financial transactions and longer running time in D2N8 than in D2N4 during one main loop of the program. One simulation system requires approximately the same transaction times to achieve an equilibrium trend.

Moreover, the aim of the LGA economic model is to simulate the income distribution. Now, let us define the income as *m* and the average income as $m_{0}$. In theory [14], the probability distribution function of individual income takes the form,

(7) $$P_{1}\left(m\right)=\frac{1}{m_{0}}\exp\left(-\frac{m}{m_{0}}\right),$$

and the income distribution function for two-earner families reads,

(8) $$P_{2}\left(m\right)=\frac{m}{m_{0}^{2}}\exp\left(-\frac{m}{m_{0}}\right).$$

**Figure 5:**
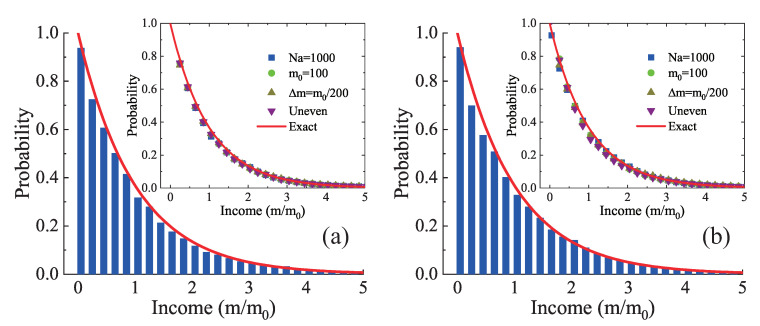
Individual income distribution: (**a**) D2N4 and exact results; and (**b**) D2N8 and exact results. The insets show the same for a different $N_{a}$, Δm, $m_{0}$, and an uneven configuration. The histogram and symbols represent simulation results, and the solid line denotes exact solutions.

Figure 5 delineates the probability distribution of individual income. The histogram represents D2N4 results in Figure 5a and D2N8 results in Figure 5b. The solid line is for the exact solutions of Equation (7). Clearly, the simulation results of D2N4 and D2N8 agree well with the exact solutions. To be specific, the income distribution has a maximum at zero income, and decreases with increasing income in a negative exponential form. Additionally, the summation of simulated income probabilities in all cases is $\sum_{m}P_{1}\left(m\right)=1$, which equals the integration of probability over all income $\int P_{1}\left(m\right)dm=1$.

Furthermore, the inserts in Figure 5 illustrate similar cases with a different parameter. The squares, circles, and upper triangles stand for the numerical simulations with $N_{a}=1000$, $m_{0}=100$, and $\Delta m=m_{0}/200$, respectively. The lower triangles are for the initial configuration with uneven distribution of money, i.e., $m_{i}=m_{0}+{\left(-1\right)}^{i}A_{m}$, with i=1, 2, ⋯, $N_{a}$. Here, the disturbance amplitude $A_{m}=5\Delta m$ is introduced to produce a non-uniform field. Clearly, for both D2N4 and D2N8, all simulation results agree well with the exact solutions. That is to say, the equilibrium distribution is independent of the number of agents, the average money, the money interchange, and the initial distribution of money. It is demonstrated that the equilibrium distribution is quite robust and does not depend on the starting conditions in the market.

**Figure 6:**
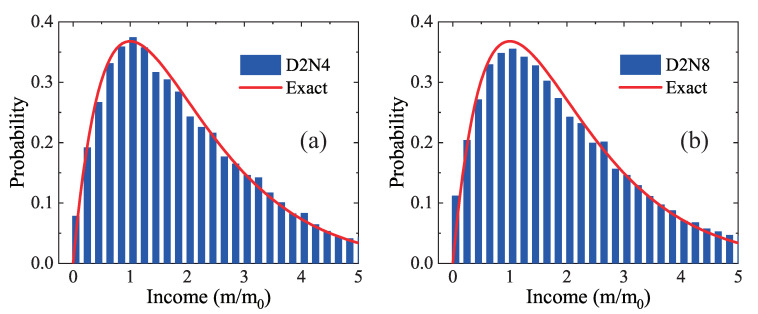
Income distribution for two-earners families: (**a**) D2N4 and exact results; and (**b**) D2N8 and exact results.

Figure 6 plots the probability distribution of family income with two earners. The histogram represents D2N4 results in Figure 6a and D2N8 results in Figure 6b. The solid line is for the exact solutions in Equation (8). There are $N_{a}/2=300$ families in the simulation program, where one agent is in random combination with another and the couple relation does not change. It can be found that both D2N4 and D2N8 results are in agreement with the exact solutions. The probability firstly increases then decreases with the increasing family income. The maximum of probability distribution is located at $m=m_{0}$. In addition, the relation $\sum_{m}P_{2}\left(m\right)=\int P_{2}\left(m\right)dm=1$ is satisfied as well.

### 3.2. Matthew Effect, Income Tax, and Charity

In the above subsection, the LGA is validated for the income distribution in the free market. In this part, the subroutines of the Matthew effect, income tax, and charity are integrated into the main program separately to test their individual impacts. Next, the three modules are considered together for the simulation of income shares in the social market.

**Figure 7:**
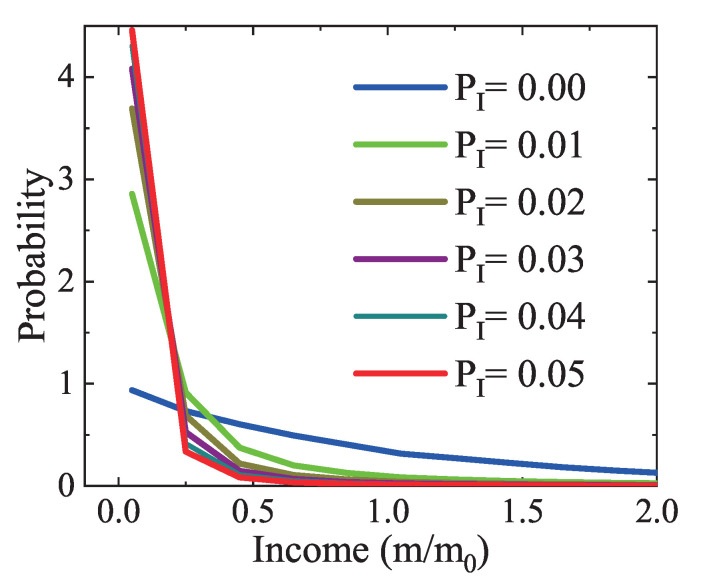
Income distributions under various inequality parameters $P_{I}=0.00$, 0.01, 0.02, 0.03, 0.04, and 0.05, respectively.

First, let us investigate the Matthew effect. Figure 7 plots the income distributions under various values of the inequality parameter from $P_{I}=0.00$ to 0.05. The case with $P_{I}=0.00$ corresponds to the one in Figure 5b. Clearly, for a larger inequality parameter, a rich (poor) agent has a larger chance to earn (loss) money, so the probability of the rich (poor) becomes smaller (larger). In other words, a few people hold more wealth, while the bottom people own fewer resources due to the Matthew effect. The computer simulations agree with the theoretical analysis. It is confirmed that the Matthew effect can be involved in the LGA.

**Figure 8:**
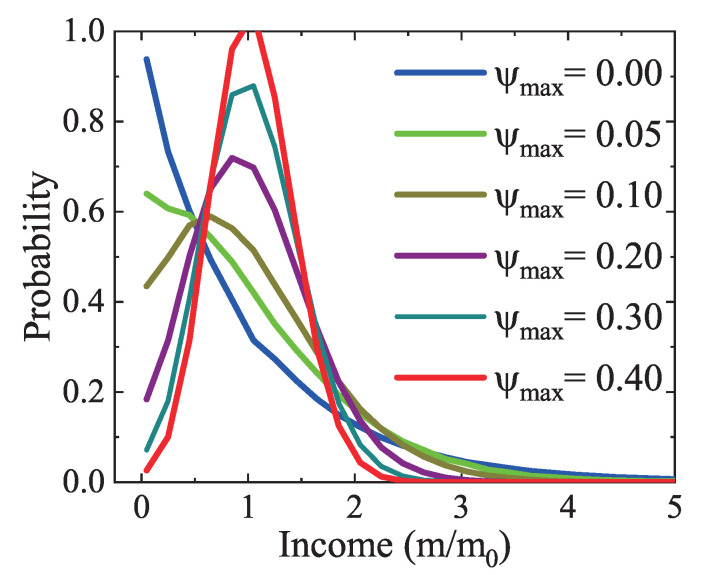
Income distributions under various maximum tax rates $\psi_{max}=0.00$, 0.05, 0.10, 0.20, 0.30, and 0.40, respectively.

Next, the influence of the income tax is studied. Figure 8 illustrates the income distributions under various tax rates. The simulation with $\psi_{max}=0$ is actually the one in Figure 5b. It can be found that, with the increasing maximum tax rate from $\psi_{max}=0$ to 0.4, both the poorer and richer populations reduce gradually, because the richer pay more taxes and the poorer draw more benefits. A peak emerges when the tax rate is large enough. The peak moves rightward and becomes higher and thinner as the tax rate increases. That is to say, there are more modest income people, and fewer people in the low- and high-income groups owing to the tax policies. The numerical results are consistent with the theoretical analysis. It is demonstrated that the income tax is incorporated correctly.

**Figure 9:**
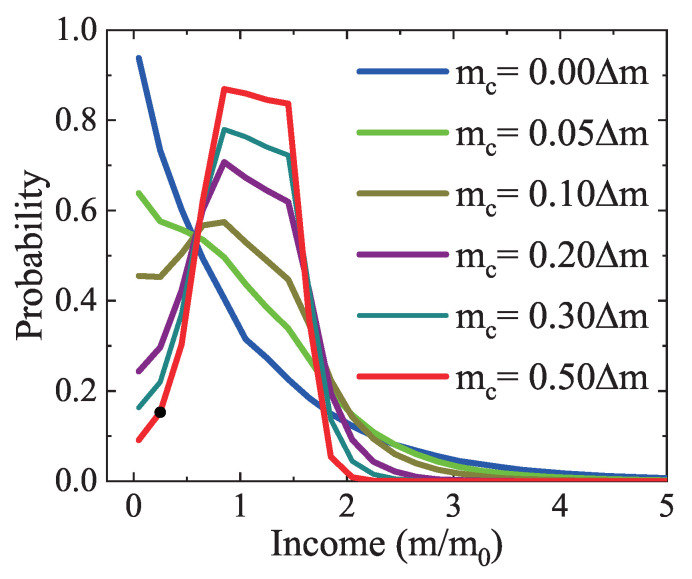
Income distributions under various charitable donations $m_{c}=0.00\Delta m$, 0.05Δm, 0.10Δm, 0.20Δm, 0.30Δm, and 0.50Δm, respectively.

Furthermore, the impact of the charity regulation is probed. Figure 9 depicts the income distributions under various charitable donations from $m_{c}=0$ to 0.5Δm. The poverty and wealthy lines are $m_{p}=0.7m_{0}$ and $m_{r}=1.5m_{0}$, respectively. The condition with $m_{c}=0$ is the same as the one in Figure 5b. Obviously, given a larger donation, the number of low- and/or high- income agents is smaller, while the density of the middle-income group becomes larger. There is a peak for a donation large enough, and the peak becomes higher and thinner with the increasing donation. Around the peak are two notable turning points at $m=m_{p}$ and $m_{r}$ due to the influence of wealthy and poverty lines. The calculation results are in line with the theoretical analysis, hence the charity is involved appropriately.

In addition, it can be found in Figure 7, Figure 8 and Figure 9 that the Matthew effect is contrary to the impact of income tax or charity. The income tax and charity play a similar role in the redistribution of wealth. It should be mentioned that the income tax refers to government regulations [53]. While it is hard to collect realistic data of the Matthew effect and charity in society, we can simulate the realistic market by adjusting the empirical parameters of the Matthew effect and charity. Despite the artificial factors, the LGA method is still helpful in evaluating relevant economic policies.

### 3.3. Income Share in the Social Market

In the realistic economic system, the Matthew effect, tax regulation, and charity play significant roles in the national income. To validate that the LGA economic model is suitable to mimic realistic markets, we consider the post-tax national income in the USA and UK, respectively [53]. Without loss of generality, the shares of total national income in the two countries in 2014 are considered as examples.

**Figure 10:**
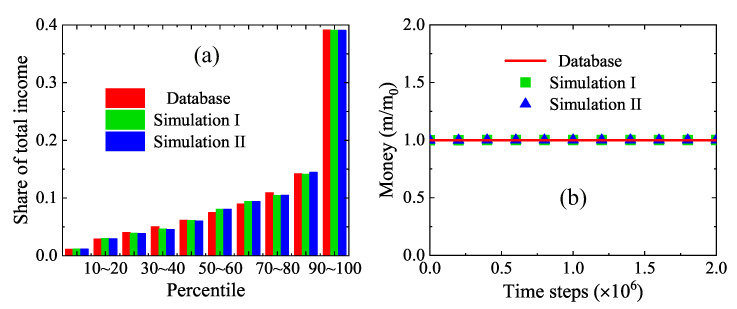
(**a**) Shares of total income in the USA in 2014: the left (red) bars denote the survey data [53], the middle (green) bars represent the simulation results with a detailed tax policy of the USA, and the right (blue) bars stand for the simulation results by using the simplified model. (**b**) Money conservation in the dynamic process: the solid line represents the exact results, the squares indicate the simulation results with a detailed tax policy of the USA, and the triangles are for the simulation results by using the simplified model.

Figure 10a displays the shares of total income in the USA in 2014. There are ten groups of shares in percentile 0∼10, 10∼20, ⋯, 90∼100, respectively. For each group, the official data [53], simulation results under the detailed income tax regulation, and simulation results from the simplified tax model are represented by the adjoining three bars from left to right, respectively. The parameters are $P_{m}=0.8$, $P_{t}=0.7$, $P_{I}=5.81\times 10^{-2}$, $m_{0}=68,844$, $m_{p}=0.4m_{0}$, $m_{r}=0.97m_{0}$, $\psi_{c}=0.35$, $m_{c}=0.5\Delta m$, $N_{a}=600$, $N_{x}\times N_{y}=50\times 50$. It is apparent that the two sets of simulation results agree well with the official data on the whole. There are slight differences between the two sets of simulations, as the simplified tax model describes an accurate relation between the tax rate and the taxable income.

To ensure the money conservation, Figure 10b plots the evolution of average individual money from zero to $2\times 10^{6}$ time steps. The solid line stands for the real average income $68,844 [53], while the squares and triangles are for the simulation results by using a detailed tax policy of the USA and the simplified model, respectively. Obviously, both sets of numerical results coincided exactly with the exact results. Consequently, it is demonstrated that the money conservation is obeyed by the LGA economic model for a realistic market.

**Figure 11:**
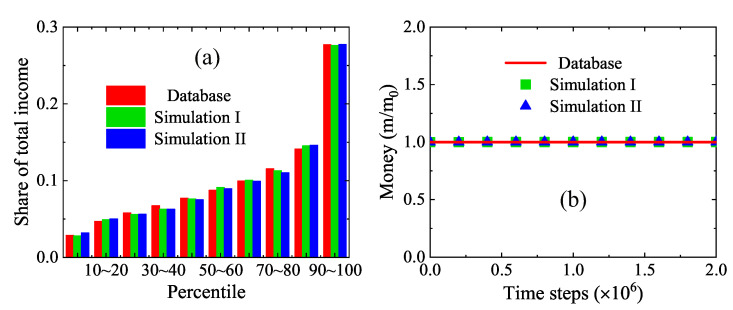
(**a**) Shares of total income in the UK in 2014: the left (red) bars denote the survey data [53], the middle (green) bars represent the simulation results with a detailed tax policy of the UK, and the right (blue) bars stand for the simulation results by using the simplified model. (**b**) Money conservation in the dynamic process: the solid line represents the exact results, the squares indicate the simulation results with a detailed tax policy of the UK, and the triangles are for the simulation results by using the simplified model.

In a similar way, Figure 11a illustrates the shares of total income in the UK in 2014. There are ten groups of shares as well. For each group, the adjoining three bars indicate the official data [53], numerical results with the detailed income tax regulation and the simplified tax model, respectively. The parameters are $P_{m}=0.8$, $P_{t}=0.7$, $P_{I}=5.74\times 10^{-2}$, $m_{0}=33,783$, $m_{p}=0.56m_{0}$, $m_{r}=m_{0}$, $\psi_{c}=0.32$, $m_{c}=0.5\Delta m$, $N_{a}=600$, $N_{x}\times N_{y}=50\times 50$. There is also a satisfactory agreement between the two runs of simulation results and survey data. Moreover, a comparison of Figure 10a and Figure 11a indicates a significant difference in the income distributions between the USA and UK because the Matthew effects, tax policies, and charitable activities are different in the two countries.

Additionally, Figure 11b shows the average individual money in the dynamic process from zero to $2\times 10^{6}$ time steps. The solid line stands for the real average income £33,783 [53], while the squares and triangles are for the simulation results by using a detailed tax policy of the UK and the simplified tax model, respectively. Clearly, the numerical results match the exact results. In fact, it is demonstrated in Figure 4, Figure 10b and Figure 11b that the money conservation is obeyed by the LGA economic model for a free or realistic market.

## 4. Conclusions and Discussion

Income inequality and financial policy have attracted intensive interest due to their great significance in human society. To this end, we present an LGA economic model for the income distribution in an ideal free market or a realistic social market. For the free market, there are two key stages, i.e., random propagation and economic transaction in the main loop of the program. We introduce two types of discrete models, i.e., D2N4 and D2N8. In the D2N4, an agent either remains motionless or travels to one of its four neighboring empty sites with a certain probability, and an economic transaction takes place randomly when two agents are located in the nearest four neighboring sites. In the D2N8, the agent may travel to one of its nearest four sites or the four diagonal sites, and exchange money with neighbors located in the four nearest or four diagonal sites randomly.

To simulate the income distribution in the social market with realistic impacts, the modules of income tax and charity are added to the phases of random propagation and economic transaction involving the Matthew effect. During the money exchange, the Matthew effect is taken into account in the way that the rich own a higher probability of earning money than the poor. Moreover, two kinds of income tax models are incorporated in the LGA economic model. One is the detailed taxable income brackets and rates, and the other is the simplified tax model based on a fitting power function. Meanwhile, charity is considered with the assumption that a richer agent donates a part of his income to charity with a given probability. Actually, there exist competitive mechanisms in the relaxation process to an equilibrium state of the income distribution. Specifically, the economic transaction leads to income differences, the Matthew effect exacerbates the disparity of wealth between the rich and poor, and the income tax and charity narrow the income gap. Meanwhile, the money conservation is obeyed due to the fact that the government should maintain money constant to avoid inflation or deflation phenomenon in the realistic society.

Finally, two types of benchmarks are used to verify and validate the current model. One is the income distributions of individual agents and two-earner families in a free market without any external factors. Both D2N4 and D2N8 are employed, and all simulations give satisfactory results of the income distribution. The other is the shares of total income in the USA and UK in 2014. Besides, impacts of the Matthew effect, income tax and charity upon the redistribution of income are investigated. It is noteworthy that, with inherent simplicity and effectiveness, the LGA economic model could be used to evaluate the influences of tax formulas (before being implemented in real society) on the income redistribution.

It should be mentioned that the consistency of numerical results with the actual data is still limited. Particularly, the model will not reproduce the detailed large-scale power-law distribution described in Ref. [15] and found in Ref. [3] because the current model is based on the assumption of the Boltzmann–Gibbs distribution, which is only approximate. In fact, the Boltzmann–Gibbs function is usually reasonable for the low and middle ranges of income distribution [14], while the Pareto function provides a good fit to the high range [15]. This is because the process of lower wealth accumulation is additive, causing a Gaussian-like distribution, while the wealth in the high class grows in a multiplicative way, generating a power-law tail [54]. To obtain more satisfactory simulation results, further research will be conducted.

## Figures and Tables

**Figure A1:**
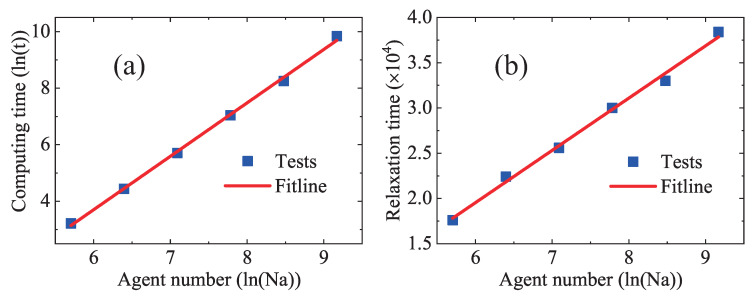
Computing time (**a**); and relaxation time (**b**) versus agent numbers. Squares represent simulation tests and solid lines indicate fitting functions.

**Figure A2:**
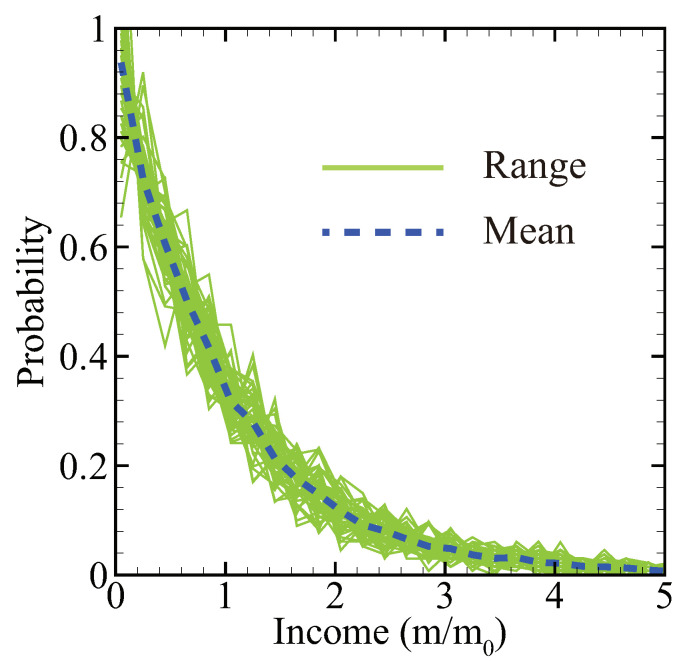
The range (solid lines) and mean (dashed line) of individual income distributions.

**Figure A3:**
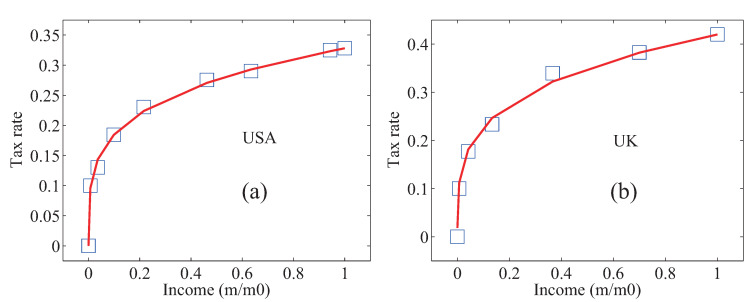
The relations between the average tax rate and the taxable income in: the USA (**a**); and the UK (**b**). The squares denote the official data and the lines stand for the fitting power functions.

## Appendix A

In an economic market with numerous agents (or a physical system consisting of massive particles), the dynamic process tends to an equilibrium state, but always in non-equilibrium states. Similarly, the simulated income distribution, which is almost always out of equilibrium, begins to oscillate around its ideal equilibrium state after an initial relaxation epoch.

In statistics, there are two ways to obtain the income distribution in the (near) equilibrium state. One method is to set the agent number $N_{a}$ and mesh grids $N_{x}\times N_{y}$ as large as possible. The income distribution of sufficient agents approaches its equilibrium counterpart after a period of relaxation process. In fact, it takes more computation time and relaxation time to obtain an equilibrium income distribution with a larger number of agents. To demonstrate it, we performed a series of numerical tests with various agent numbers. (The mesh grids $N_{x}\times N_{y}$ are proportional to the agent number.) Other parameters are the same as those in Figure 4. As shown in Figure A1a, the computing time $t_{c}$ (seconds) increases with the increasing agent number, and the relation between them is described by a fitting function $\ln\left(t_{c}\right)=1.9\times \ln\left(N_{a}\right)-7.6$. Here, the computing time $t_{c}$ is taken by the program running for $2\times 10^{5}$ time steps. Figure A1b depicts that the relaxation time $t_{r}$ (steps) increase with the increasing agent number, and the fitting function is $t_{r}=0.58\times \ln\left(N_{a}\right)-1.5$. Clearly, it demands much longer calculation time and higher computational cost to mimic an economic system with more agents.

The other method is to take the average of simulated income distributions at various time instants after the initial relaxation process. In Figure A2, the solid lines represent the probability distributions of individual income from time instants $t=10^{5}$ to $2\times 10^{5}$ with interval of 2000 time steps, and the dashed line denotes the mean. Actually, the dashed line in Figure A2 corresponds to the histogram in Figure 5a. It is noteworthy that this method is more efficient than the former one. All equilibrium income distributions are obtained with this time-average method in this work.

## Appendix B

The detailed income tax brackets and rates of the USA [55] and UK [56] are shown in Table A1 and Table A2, respectively. In each table, the left column lists the income range and the right column gives the corresponding marginal tax rate. There are seven levels of tax rates in the USA in Table A1 and four levels in the UK in Table A2. For a higher taxable income bracket, the marginal tax rate is higher in both the USA and UK.

**Table A1:** Taxable income brackets and rates of the USA in 2014.

Income (Dollar)	Marginal Tax Rate
$0–9075	10%
$9076–36,900	15%
$36,901–89,350	25%
$89,351–186,350	28%
$186,351–405,100	33%
$405,101–406,750	35%
≥$406,751	39.6%

**Table A2:** Taxable income brackets and rates of the UK in 2014.

Income (GBP)	Marginal Tax Rate
£0–2880	10%
£2881–31,865	20%
£31,866–150,000	40%
≥£150,000	45%

In this work, a simplified tax model is proposed to describe the relationship between the taxable income and the average tax rate. To be specific, an average tax rate is the ratio of the total tax liability to the total tax base, which can be calculated from the information in Table A1 or Table A2. Then, a fitting function could be obtained for the relationship between the income and the average tax rate. Figure A3 delineates the average tax rate ψ versus the taxable income *m*. In Figure A3a, the squares denote the data of the USA, and the line is for the fitting function $\psi =0.328{\left(m/m_{max}\right)}^{0.249}$ with $m_{max}=\$640,000$. In Figure A3b, the squares denote the data of the UK, and the line is for the fitting function $\psi =0.42{\left(m/m_{max}\right)}^{0.264}$ with $m_{max}=£300,000$. Obviously, the fitting functions agree well with the official data of the USA and UK, respectively. Hence, the simplified tax model based on the fitting function can be substituted for the detailed income tax brackets and rates.
